# Precision-cut tumor slices for modeling hepatocellular carcinoma enable at-scale drug screening

**DOI:** 10.1097/HC9.0000000000000706

**Published:** 2025-05-16

**Authors:** Amy L Collins, Keara Kirkness, Erik Ramon-Gil, Eleni Tzortzopoulou, Daniel Geh, Jack Dishington, Eleanor Graham, Rhys Muir, Rainie Cameron, Saimir Luli, Eman Khurram, Daniel Storey, Hannah L. Paish, Glyn Nelson, David McDonald, Andrew Filby, Lee A. Borthwick, Fiona Oakley, Derek A. Mann, Jack Leslie

**Affiliations:** 1 Newcastle Fibrosis Research Group, Biosciences Institute, Faculty of Medical Sciences, Newcastle University, Newcastle upon Tyne, UK; 2 The Newcastle University Centre for Cancer, Newcastle University, Newcastle upon Tyne, UK; 3 Newcastle University Medicine Malaysia, Iskandar Puteri, Malaysia; 4 FibroFind Ltd, William Leech Building, Medical School, Newcastle University, Newcastle upon Tyne, UK; 5 Bioimaging Unit, Newcastle University, Newcastle upon Tyne, UK; 6 Flow Cytometry Core Facility, Biosciences Institute, Newcastle University, Newcastle upon Tyne, UK; 7 Department of Gastroenterology and Hepatology, School of Medicine, Koç University, Istanbul, Turkey

**Keywords:** cancer, model, screening

## Abstract

**Background:**

Disease modeling is vital for our understanding of disease mechanisms and for developing new therapeutic strategies. Accurately modeling the intact tumor microenvironment (TME) is increasingly recognized as essential for gaining insights into cancer biology and therapeutic response. Preclinical mouse models have provided utility for studying the evolving TME, but these models are costly and can lead to animal suffering and the discontinuation of drug investigations. To address these limitations, particularly in hepatocellular carcinoma (﻿HCC), we have developed an ex vivo model using tumor precision-cut slices (TPCS) derived from orthotopic liver tumors.

**Methods:**

Murine HCC tumors were generated via intrahepatic injection of Hep-53.4 cells, providing a source of tumor tissue for TPCS generation. Subsequent scaling to a 96-well format and modification to include a secreted luciferase enabled longitudinal ex vivo screening of 26 drugs applied at 2 doses over an 8-day period, using just 5 tumors. One drug identified in the screen, salinomycin, was then validated in vivo via intraperitoneal injection of mice with orthotopic liver tumors.

**Results:**

Histological characterization determined that TPCS maintain the architecture, cellular complexity, and drug responsiveness of the original HCC-TME under simplified culture conditions that preserve viability and metabolic activity. In addition to typical HCC therapies, sorafenib and anti-PD1 immunotherapy, the screen identified 2 drugs as potent anticancer agents capable of impacting the viability of TPCS: salinomycin and rottlerin. Salinomycin was further validated in vivo, significantly reducing tumor burden without evidence of toxicity.

**Conclusions:**

We present a 3Rs (Reduction, Refinement, Replacement) approach for studying HCC biology and performing 96-well-scale drug screening within an intact, metabolically active TME, offering a more ethical and effective platform for drug discovery.

## INTRODUCTION

Liver cancer presents a growing global health challenge and is one of the few cancer types increasing in incidence. There were ~906,000 new cases of liver cancer in 2020, and hepatocellular carcinoma (HCC) comprises roughly 80% of primary liver cancer cases.^1^^,^^2^ A number of risk factors are associated with the development of HCC, including viral infection with hepatitis B and hepatitis C, metabolic dysfunction–associated steatotic liver disease (MASLD), metabolic–associated steatohepatitis (MASH), and alcohol-associated liver disease (ALD).^3^^,^^4^ The majority of HCC cases are attributed to chronic liver disease, and cirrhosis is the strongest risk factor for HCC development.^5^^,^^6^

Despite the recent emergence of novel therapeutic options for the treatment of advanced HCC, efficacy is limited, and benefits are limited to a minority of patients. A combination of atezolizumab (anti-PD-L1) and bevacizumab (anti-VEGF) was demonstrated to be superior to the previous gold standard therapy for advanced HCC, the multi-target tyrosine kinase inhibitor sorafenib.^7^ However, the overall survival benefit offered by this combination therapy is less than 14 months, and roughly 30% of patients exhibit a therapeutic response. Superior therapeutic strategies are required to target highly aggressive tumors to overcome the limited efficacy of current therapeutics if we are to improve patient outcomes.^8^

The tumor microenvironment (TME) is a complex ecosystem in which crosstalk between various cellular components contributes to HCC evolution and therapeutic responses.^9^ HCC animal models are considered to be effective preclinical platforms in which all components of the TME are accounted for. Orthotopic xenograft mouse models of HCC generate fast-growing tumors suitable for the screening of novel therapies, and it has previously been demonstrated that Hep-53.4 cells implanted into the mouse liver grow and evolve fibrotic tumors with an abundance of infiltrating T cells and dendritic cells, and are responsive to anti-PD1 immunotherapy.^10^^,^^11^ Furthermore, transcriptomic analysis identified that Hep-53.4 orthotopic tumors are both poorly differentiated and highly proliferative, indicating the model offers utility for therapeutic screening for aggressive HCC tumors.^12^

Precision-cut liver slices and tumor precision-cut slices (PCLS and TPCS, respectively) retain the architecture, ECM composition, and complex cell/cell interactions of a native tissue and provide a platform for studying disease mechanisms and therapeutic response ex vivo.^13^^–^^15^ Routinely acquiring large quantities of human tumor tissue for the generation of TPCS can present difficulties, given the diagnostic and prognostic importance of resected tumor samples, which must be carefully examined by pathologists so as not to compromise patient care. Additionally, the degree of structural and cellular heterogeneity between different patient tumor samples, and even within any one individual HCC tumor, can be considerable and therefore makes standardization of the platform challenging, this being particularly problematic for oncology drug screening and development. As a result, we sought to generate TPCS from orthotopic murine Hep-53.4 tumors to provide a robust, reproducible ex vivo model of HCC capable of assessing novel therapeutic strategies to target poorly differentiated HCC. The ability to screen multiple therapeutic combinations in TPCS from the same tumor provides a cost-effective method of assessing drug efficacy in line with the 3Rs: reducing, replacing and refining the use of animals in research.

## METHODS

### Mice

All animal experiments were approved by the Newcastle Ethical Review Committee and performed under a UK Home Office licence, in accordance with the ARRIVE guidelines. All mice were housed in the Comparative Biology Centre at Newcastle University in pathogen-free conditions with free access to food and water. Male C57BL/6 wild-type (WT) mice were purchased from Envigo.

### Cell culture

Hep-53.4 and Hepa1-6 cells were cultured in DMEM with high glucose (Sigma-Aldrich), and H22 cells were cultured in RPMI-1640 (Sigma-Aldrich), at 37 °C with 5% CO_2_. Culture media was supplemented with 10% FBS, 1% penicillin–streptomycin, 1% l-glutamine, and 1% pyruvate. Cells were routinely screened for mycoplasma and were mycoplasma-negative. To screen a panel of 26 compounds, 5000 Hep-53.4 cells were seeded per well in 96-well culture plates and treated with compounds or controls for 4 days.

### Orthotopic liver cancer model

Surgeries were performed under general anesthesia with isoflurane and all animals were administered with pre-surgery and post-surgery buprenorphine (0.003 mg/mL). Following a laparotomy, 1×10^6^ Hep-53.4, Hepa1-6, or H22 HCC cells (Cytion) resuspended in 20 μL of 30% Matrigel in sterile PBS (v/v) were implanted into the large lobe of male C57BL/6 mice via intrahepatic injection. In vivo imaging systems (IVIS) imaging was performed weekly to assess tumor growth via bioluminescence. Mice with Hep-53.4 tumors were harvested at days 14, 21, and 28 for characterization of tumor and liver tissue. Therapeutic intervention with 45 mg/kg sorafenib (Tocris), 10 mg/kg lenvatinib (Selleckchem), or PEG/DMSO control was administered via daily oral gavage from day 14 until mice were harvested at day 28. Therapeutic intervention with 4 mg/kg salinomycin (MedChemExpress) or a corn oil/DMSO control was administered via daily intraperitoneal injection from day 14 until mice were harvested at day 28.

### Precision-cut liver and tumor slice generation

Liver and tumor tissue were cored using a 3 mm or 8 mm Stiefel biopsy punch (Medisave). Tissue cores were submerged in 3% low-gelling temperature agarose (Sigma-Aldrich). Agarose-embedded tissue cores were cut using a Leica VT1200S vibrating blade microtome (Leica) at a depth of 250 μm. PCLS and TPCS generated were cultured in 8 µm-pore Transwell inserts in our Bioreactor as previously described,^14^ or alternatively in static 12-well or 96-well culture plates. Tissue was cultured in Williams’ Medium E (Sigma-Aldrich) supplemented with 1% penicillin–streptomycin, 1% l-glutamine, 1% pyruvate, 1× insulin transferrin-selenium X, 2% fetal bovine serum, and 100 nM dexamethasone, at 37 °C supplemented with 5% CO_2_. Media was completely replaced daily.

### Spheroid generation

Spheroids were formed by seeding 10,000 Hep-53.4 cells per well in agarose-coated round-bottom 96-well plates in a total volume of 100 μL and incubating at 37 °C with 5% CO_2_. To screen a panel of 26 compounds, Hep-53.4 spheroids were treated with each compound or control from day 1 to day 4 with complete media changes daily. Spheroids were imaged using a Zeiss AXIO Observer D1 microscope, and spheroid measurements were recorded using Zen (blue edition) software.

See Supplemental Digital Content (SDC, Supplemental Digital Content 1, http://links.lww.com/HC9/B979) for additional methods.

## RESULTS

### Characterization of an orthotopic HCC model for TPCS production and preclinical drug testing

**FIGURE 1:**
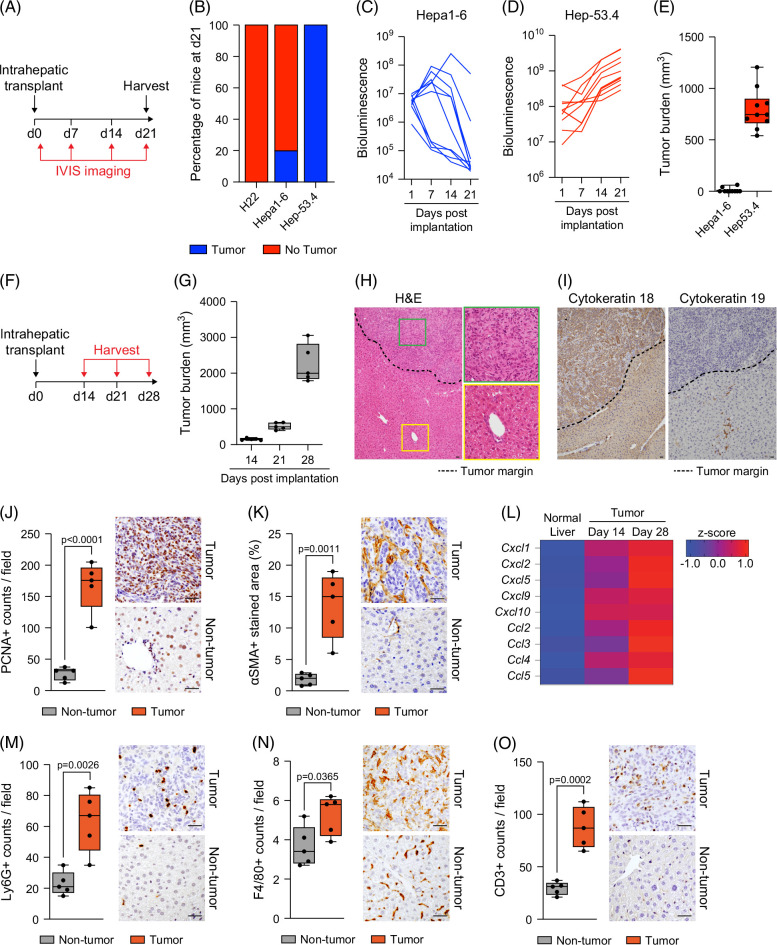
Hep-53.4 cells generate large, fast-growing orthotopic tumors. (A) Schematic shows the timeline of intrahepatic transplant of Hep-53.4, Hepa1-6, or H22 cells and subsequent weekly IVIS imaging to assess HCC engraftment and growth. (B) Percentage stacked bar chart shows the percentage of mice that developed tumors 21 days after intrahepatic transplant of Hep-53.4, Hepa1-6, or H22 cells. N=10 mice per cell line. (C, D) Graphs show bioluminescence levels from IVIS imaging of mice at 1, 7, 14, and 21 days following intrahepatic injection of (C) Hepa1-6 or (D) Hep-53.4 cells. (E) The graph shows tumor burden of Hepa1-6 and Hep-53.4 orthotopic tumors 21 days after intrahepatic transplant. Data are mean ± SEM from N=10 mice per cell line. (F) Schematic shows the timeline of intrahepatic transplant of Hep-53.4 cells and subsequent harvest at days 14, 21, and 28. (G) The graph shows tumor burden of Hep-53.4 tumors harvested 14, 21, and 28 days after intrahepatic transplant. Data are mean ± SEM from N=5 mice per time point. (H, I) Representative images of (H) H&E-stained and (I) cytokeratin-18-stained and cytokeratin-19-stained tumor and non-tumor tissue. Black dotted line denotes tumor margin. (J, K) Histological quantification and representative images of (J) PCNA-stained and (K) αSMA-stained tumor and non-tumor tissue harvested 28 days after intrahepatic transplant. Data are mean ± SEM from N=5 mice. (L) Heatmap showing expression of *Cxcl1*, *Cxcl2*, *Cxcl5*, *Cxcl9*, *Cxcl10*, *Ccl2*, *Ccl3*, *Ccl4*, and *Ccl5* in normal liver tissue and Hep-53.4 tumor tissue 14 and 28 days after intrahepatic transplant. (M–O) Histological quantification and representative images of (M) Ly6G-stained, (N) F4/80-stained, and (O) CD3-stained tumor and non-tumor tissue harvested 28 days after intrahepatic transplant. Data are mean ± SEM from n = 5 mice. Scale bars: 50 μm. Abbreviations: H&E, hematoxylin and eosin; IVIS, in vivo imaging systems.

Given the challenges in acquiring large quantities of fresh HCC tumor resection material suitable for generating TPCS, we developed a rapid syngeneic orthotopic HCC model for excision of tumors for precision slicing. To establish the model, we identified 3 commercially available murine cell lines of hepatocellular origin, luciferase-tagged them, and intrahepatically injected 1×10^6^ cells into the large lobe of the liver. Tumor engraftment and growth were monitored weekly by bioluminescence IVIS imaging (Figure 1A). Only Hep-53.4 cells resulted in 100% engraftment, an exponential increase in bioluminescence signal, and the reproducible formation of large macroscopic tumors (Figures 1B–E).

We next determined the kinetics and humane endpoints of macroscopic tumor development in the Hep-53.4 model. Mice received a single intrahepatic injection of 1×10^6^ Hep-53.4 cells and were harvested weekly (Figure 1F). Small macroscopic tumors were observed at 14 days post-injection (Figure 1G). Tumor growth was exponential, increasing significantly between day 21 and day 28, after which animals were typically humanely killed due to tumor burden (Figure 1G). Histological analysis of the Hep-53.4 tumors revealed localization of the tumor primarily to one half of the left lobe, with thickening of the hepatic plate being visible (Figure 1H). Immunohistochemical (IHC) staining for cytokeratin-18 revealed that Hep-53.4 tumors are of hepatocellular origin and were not positive for the biliary marker cytokeratin-19 (Figure 1I). IHC for PCNA and αSMA revealed that Hep-53.4 tumors are highly proliferative and include a significant population of activated cancer-associated fibroblasts (Figures 1J, K).

We have previously shown by whole-exome sequencing that Hep-53.4 cells are mutant for p53 and WT for CTNNB1, as well as APC and AXIN2, suggesting these tumors were likely to be immunogenic and would potentially respond to immunotherapy.^16^ Analysis of Hep-53.4 tumors revealed they were highly immunogenic, displaying increased proinflammatory gene expression (Figure 1L) and increased numbers of infiltrating Ly6G^+^ neutrophils, F4/80^+^ macrophages, and CD3^+^ T cells compared to adjacent normal liver (Figures 1M–O).

### TPCS retain characteristics of in vivo tumors in culture

**FIGURE 2:**
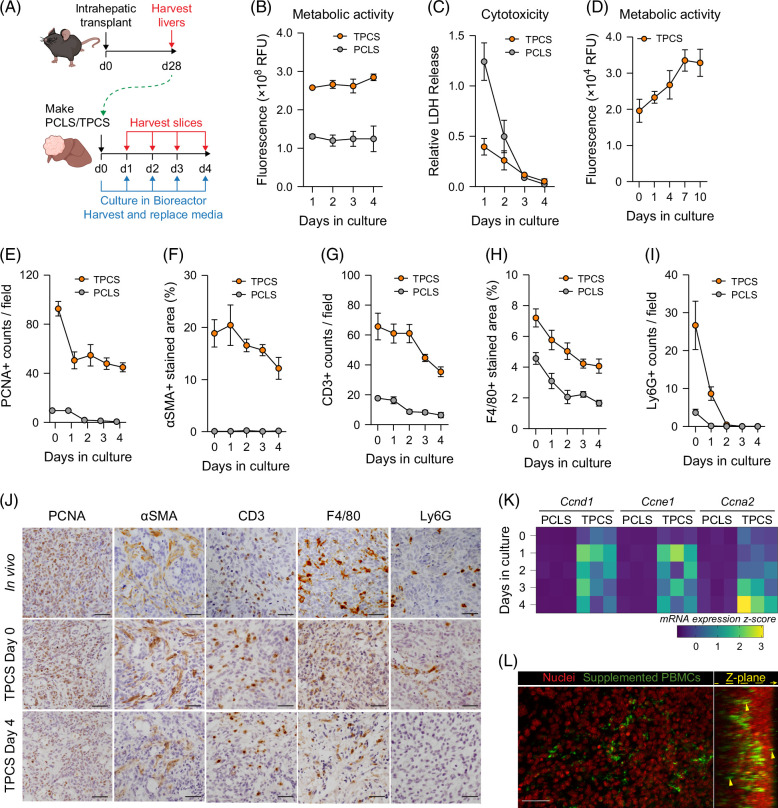
TPCS retain the characteristics of the original tumor in culture. (A) Schematic shows the timeline of intrahepatic transplant of Hep-53.4 cells to generate orthotopic tumors for the generation of TPCS and PCLS, and a subsequent 4-day culture period in a rocked Bioreactor platform. (B, C) Graphs show levels of (B) metabolic activity measured via resazurin assay and (C) cytotoxicity measured via LDH assay from TPCS and PCLS cultured for 4 days in a rocked Bioreactor platform. Data are mean ± SEM from N=3 TPCS/PCLS. (D) The graph shows metabolic activity measured via resazurin assay of TPCS cultured for 10 days in a rocked Bioreactor platform. Data are mean ± SEM from up to N=6 TPCS per time point. (E-I) Histological quantification of (E) PCNA-stained, (F) αSMA-stained, (G) CD3-stained, (H) F4/80-stained, and (I) Ly6G-stained TPCS and PCLS cultured for 4 days in a rocked Bioreactor platform. Data are mean ± SEM from N=3 TPCS/PCLS per time point. (J) Representative images of in vivo tumors and TPCS at day 0 and day 4 stained for PCNA, αSMA, CD3, F4/80, and Ly6G. Scale bars: 50 μm. (K) Heatmap showing expression of cell cycle genes *Ccnd1*, *Ccne1*, and *Ccna2* in PCLS and TPCS between 0 and 4 days in culture. (L) Multiphoton image shows z-plane of a TPCS stained with Hoechst (red) following the addition of murine peripheral blood mononuclear cells (green) to the culture 1 day prior. Abbreviations: LDH, lactate dehydrogenase; PCLS, precision-cut liver slice; TPCS, tumor precision-cut slices.

Human PCLS are an ex vivo culture system used to study hepatic fibrosis and drug metabolism.^14^^,^^15^^,^^17^ To investigate the potential for this type of preclinical platform to be applied to ex vivo mouse tumors, we generated PCLS and TPCS from our highly characterized HCC mouse model and cultured them in a previously described bioreactor system that extends the viability of PCLS (Figure 2A).^14^ Like human and rat PCLS,^14^ mouse PCLS and TPCS cultured in the rocked bioreactor system were metabolically active following 4 days in culture (Figure 2B). Analysis of lactate dehydrogenase (﻿LDH), indicative of necrosis, showed that cell death resultant of tissue slicing began to stabilize after 24 hours in culture, and of note we observed lower levels of cell death in TPCS compared with PCLS (Figure 2C). Tissue morphology and integrity, evaluated by H&E staining, was maintained up to day 4 in Bioreactor-cultured PCLS and TPCS (Supplemental Figure S2A, Supplemental Digital Content 1, http://links.lww.com/HC9/B979). However, mouse PCLS viability began to deteriorate after 3 days in culture (Supplemental Figure S2B, Supplemental Digital Content 1, http://links.lww.com/HC9/B979 and Supplemental Figure S5C, Supplemental Digital Content 1, http://links.lww.com/HC9/B979), possibly due to the fragility of the tissue, this contrasting with stable viability for up to 6 days for rat and human PCLS maintained in the bioreactor.^14^ Conversely, TPCS remained metabolically active for at least 10 days with no obvious necrosis despite the increased cellular density present in TPCS compared to PCLS (Figure 2D).

TPCS cultured for 4 days maintained an elevated number of PCNA^+^ tumor cells, an αSMA^+^ stroma, and maintenance of both CD3^+^ T cells and F4/80^+^ macrophages when cultured for 4 days, albeit with a modest reduction in their numbers being observed after 2 days (Figures 2E–H). By contrast, Ly6G^+^ neutrophils were present in the tissue for only the first 24 hours of culture due to their short lifespan (Figure 2I). With the exception of neutrophils, TPCS cultured ex vivo maintained the phenotype of the original tumors (Figure 2J and Supplemental Figure S2C, Supplemental Digital Content 1, http://links.lww.com/HC9/B979). Additionally, TPCS displayed increased expression of cell cycle genes compared to non-tumor PCLS (Figure 2K). Whilst TPCS do not account for the recruitment of immune cells, supplementing ex vivo cultured TPCS with peripheral blood mononuclear cells (PBMCs) demonstrated that immune cells added to the culture system successfully invade the tumor tissue (Figure 2L and Supplemental Movie S1). Taken together, these observations suggest that bioreactor-cultured TPCS represent a potential ex vivo platform for studying an intact metabolically active TME.

### TPCS recapitulate in vivo responses to HCC systemic therapies

**FIGURE 3:**
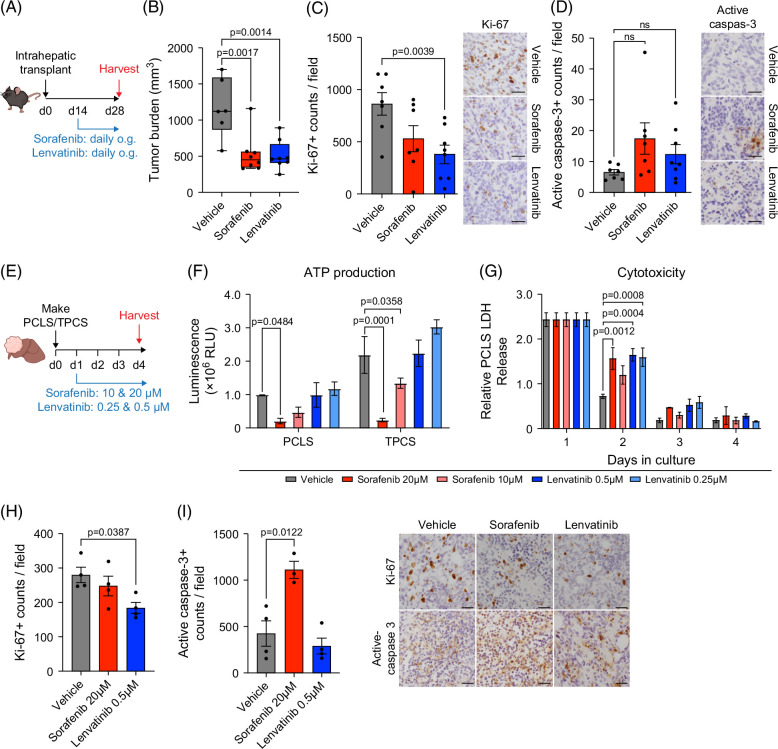
TPCS recapitulate in vivo responses to HCC tyrosine kinase inhibitors. (A) Schematic shows the timeline of intrahepatic transplant of Hep-53.4 cells to generate orthotopic tumors, followed by therapeutic intervention at day 14 with sorafenib (45 mg/kg), lenvatinib (10 mg/kg), or vehicle control via daily oral gavage before tumors were harvested at day 28. (B) The graph shows tumor burden of Hep-53.4 orthotopic tumors treated with vehicle control, sorafenib, or lenvatinib. Data are mean ± SEM from up to N=8 mice per treatment group. (C, D) Graphs show histological quantification and representative images of Hep-53.4 tumors stained for (C) Ki-67 and (D) active caspase-3 from mice treated with vehicle control, sorafenib, or lenvatinib. Data are mean ± SEM from up to N=8 mice per treatment group. (E) Schematic shows the timeline of TPCS/PCLS generation and subsequent treatment with vehicle control, sorafenib (10–20 µM) or lenvatinib (0.25–0.5 µM) throughout a 4-day period in a rocked Bioreactor platform. (F) The graph shows ATP production measured via CellTiter-Glo assay from PCLS and TPCS harvested at day 4 following culture with vehicle control, sorafenib (10–20 µM) or lenvatinib (0.25–0.5 µM). Data are mean ± SEM from N=2 PCLS/TPCS. (G) The graph shows cytotoxicity measured via LDH assay from PCLS treated with vehicle control, sorafenib (10–20 µM) or lenvatinib (0.25–0.5 µM) across 4 days in culture. Data are mean ± SEM from N=2 PCLS. (H, I) Histological quantification and representative images of (H) Ki-67-stained and (I) active capsase-3–stained TPCS following treatment with vehicle control, sorafenib (20 µM) or lenvatinib (0.5 µM) across 4 days in culture. Data are mean ± SEM from up to N=4 TPCS. Scale bars: 50 μm. Abbreviations: LDH, lactate dehydrogenase; PCLS, precision-cut liver slice; TPCS, tumor precision-cut slices.

We next asked if orthotopic HCC responds to the tyrosine kinase inhibitors (TKIs) sorafenib and lenvatinib, which are both first-line treatments for advanced HCC (Figure 3A). Sorafenib targets serine/threonine and tyrosine kinases in multiple oncogenic and angiogenic signaling pathways, resulting in its well characterized cytostatic, apoptotic and anti-angiogenic effects.^18^^,^^19^ Lenvatinib on the other hand is primarily thought to enact its antitumor effects by suppressing angiogenesis via targeting VEGFR1-3, although cytostatic effects have been described in vitro.^20^ Compared to vehicle control, sorafenib-treated and lenvatinib-treated mice displayed a significant reduction in tumor burden and liver weight (Figure 3B and Supplemental Figure S3A, Supplemental Digital Content 1, http://links.lww.com/HC9/B979). This therapeutic effect was associated with a significant reduction in proliferating Ki-67^+^ cells in lenvatinib-treated mice and a nonsignificant increase in active caspase-3^+^ apoptotic tumor cells in sorafenib-treated mice (Figures 3C, D).

To determine whether these cytostatic and apoptotic effects observed in vivo could be recapitulated ex vivo, PCLS and TPCS were generated from livers with orthotopic Hep-53.4 tumors and treated with sorafenib and lenvatinib (Figure 3E). Daily resazurin and CellTiter-Glo assays were performed on TPCS and PCLS to assess metabolic activity and ATP production, respectively, while LDH levels were measured to examine possible cytotoxicity in non-tumor liver tissue following drug treatment. In line with the in vivo TKI responses, treatment of TPCS with 20 μM of sorafenib resulted in a significant decrease in tumor slice viability characterized by a reduction in both ATP production and metabolic activity (Figure 3F and Supplemental Figure S3B, Supplemental Digital Content 1, http://links.lww.com/HC9/B979), while analysis of LDH release demonstrated that 20 μM sorafenib was associated with hepatotoxicity in the initial 24 hours of treatment before this effect ameliorated (Figure 3G). Additionally, whilst no significant change was observed in cancer cell proliferation in the TPCS, an increase in active caspase-3^+^ apoptotic cells was observed following treatment with 20 μM sorafenib, reflective of the in vivo immunohistochemical data (Figures 3H, I). These data suggest that the Hep-53.4 TPCS/PCLS system can be used to identify drug-related toxicities, whereby efficacious drug concentrations that are critically nontoxic can be identified.

In contrast to sorafenib, TPCS treated with lenvatinib showed no decrease in tumor cell or hepatocyte viability (Figure 3F and Supplemental Figure S3B, Supplemental Digital Content 1, http://links.lww.com/HC9/B979). Hepatotoxicity was observed in PCLS during the initial 24 hours of treatment only, before LDH release abated for the remainder of the culture period, and there was no significant impact on PCLS metabolic activity (Figure 3G and Supplemental Figure S3C, Supplemental Digital Content 1, http://links.lww.com/HC9/B979). Mirroring the in vivo immunohistochemical findings, lenvatinib treatment was associated with a decrease in Ki-67^+^ proliferative cells whilst causing no change in apoptosis in TPCS (Figures 3H, I). Taken together these data suggest that the TPCS system largely recapitulates the TKI therapy responses observed in the in vivo orthotopic HCC model.

**FIGURE 4:**
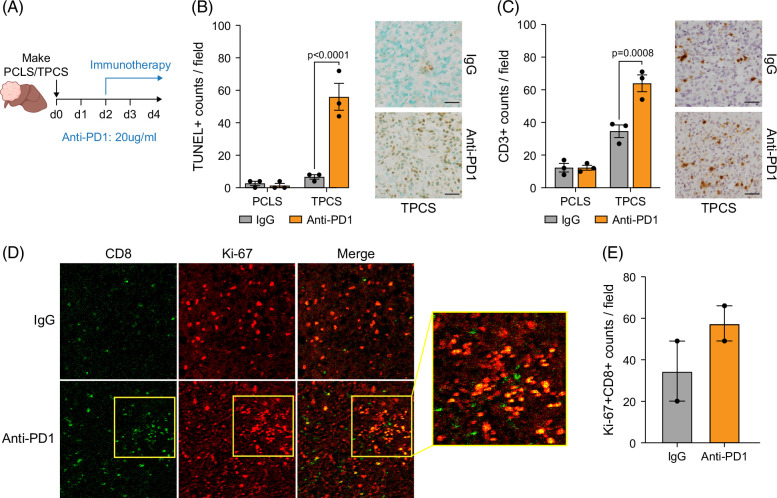
TPCS recapitulate in vivo responses to immunotherapy. (A) Schematic shows the timeline of TPCS/PCLS generation and subsequent 4-day culture period, with therapeutic intervention with anti-PD1 (20 µg/mL) from day 2 onwards. (B, C) Histological quantification and representative images of (B) TUNEL-stained and (C) CD3-stained PCLS and TPCS following culture with isotype control or anti-PD1 (20 µg/mL). Data are mean ± SEM from N=3 PCLS/TPCS per treatment group. (D) Representative images from Hyperion multiplex spatial analysis of TPCS cultured with isotype control or anti-PD1 (20 µg/mL), showing CD8 (green) and Ki-67 (red). (E) The graph shows quantification of Ki-67^+^CD8^+^ T cells from Hyperion imaging mass cytometry images. Data are mean ± SEM for N=2 regions of interest. Scale bars: 50 μm. Abbreviations: PCLS, precision-cut liver slice; TPCS, tumor precision-cut slices.

We previously reported that Hep-53.4 orthotopic tumors are responsive to anti-PD1 immunotherapy.^16^ We therefore next determined whether TPCS from orthotopic tumors would also respond to immunotherapy (Figure 4A). Compared to IgG-treated controls, anti-PD1-treated TPCS displayed increased cell death characterized by elevated TUNEL staining (Figure 4B). Importantly, this effect was limited to TPCS, with anti-PD1-treated PCLS having comparable levels of TUNEL staining to IgG controls (Figure 4B). In line with the published in vivo model, there was an associated significant increase in numbers of CD3+ T cells in anti-PD1-treated TPCS (Figure 4C). To determine whether T cells were actively proliferating in the anti-PD1-treated TPCS, a multiplexed spatial analysis was performed. This revealed that anti-PD1 immune checkpoint blockade promotes CD8^+^ T cell proliferation as identified by dual Ki-67^+^CD8^+^ positive T cells (Figures 4D, E).

Taken together these data demonstrate that TPCS can effectively maintain the TME in culture and recapitulate both TKI and immunotherapy treatment effects observed in the in vivo model, therefore offering an ex vivo murine preclinical HCC platform for studying therapeutic responses.

### Modeling tumor evolution and miniaturizing the TPCS platform

**FIGURE 5:**
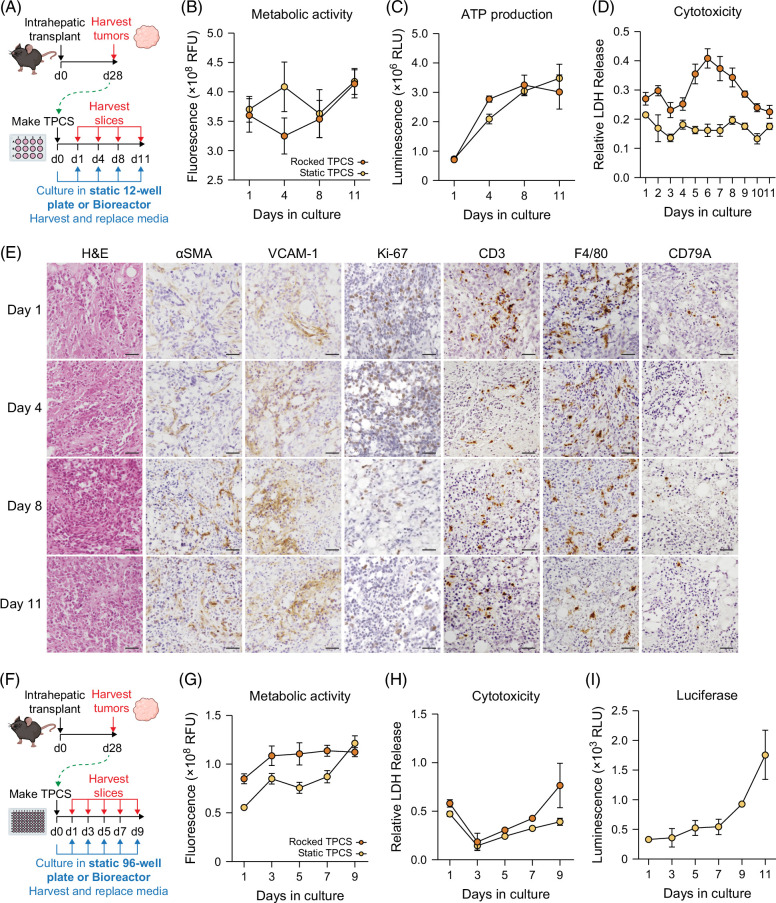
Modeling tumor evolution and miniaturization of TPCS. (A) Schematic shows the timeline of intrahepatic transplant of Hep-53.4 cells to generate orthotopic tumors for TPCS generation, and subsequent 11-day culture period in static 12-well culture plates or the rocked Bioreactor platform. (B–D) Graphs show (B) metabolic activity, (C) ATP production, and (D) cytotoxicity from TPCS cultured for 11 days in 12-well static culture or a rocked Bioreactor platform, measured via resazurin assay, CellTiter-Glo assay, and LDH assay, respectively. Data are mean ± SEM from N=5 TPCS per group and time point. (E) Representative images of TPCS cultured in static plates at days 1, 4, 8, and 11. TPCS were H&E-stained and immunohistochemically stained for αSMA, VCAM-1, Ki-67, CD3, F4/80, and CD79A. (F) Schematic shows the timeline of intrahepatic transplant of Hep-53.4 cells to generate orthotopic tumors for the generation of miniaturized 3 mm TPCS, and subsequent 9-day culture period in static 96-well culture plates or the rocked Bioreactor platform. (G, H) Graphs show (G) metabolic activity and (H) cytotoxicity from TPCS cultured for 9 days in 96-well static culture or a rocked Bioreactor platform, measured via resazurin assay and LDH assay, respectively. Data are mean ± SEM from N=4 TPCS per group. (I) The graph shows longitudinal luciferase assay data from SecLuc TPCS cultured in static 96-well culture plates for 11 days. Data are mean ± SEM from N=4 TPCS. Scale bars: 50 μm. Abbreviations: H&E, hematoxylin and eosin; LDH, lactate dehydrogenase; TPCS, tumor precision-cut slices.

Given the observed robustness of ex vivo cultured TPCS, we hypothesized that as tumor slices remain viable for longer than liver slices, the former may not require the bioreactor system, potentially simplifying the model to make it accessible to investigators lacking access to the bioreactor. We therefore longitudinally assessed the viability of TPCS cultured in both the Bioreactor and standard static 12-well plates (Figure 5A). The data collected indicated that unlike PCLS, TPCS can be cultured in standard 12-well plates without any detrimental effect on slice viability, and with no significant differences observed in metabolic activity or ATP production between rocked and static TPCS (Figures 5B, C). Unexpectedly, TPCS cultured in static 12-well plates released lower levels of LDH than rocked TPCS in the Bioreactor, indicative of lower cytotoxicity, which provides a significant advantage of avoiding rocking the tissue (Figure 5D).

To determine how the TME evolves over the extended static culture period, harvested TPCS were H&E-stained and immunohistochemically analyzed for Ki-67, αSMA, VCAM-1, CD3, F4/80 and CD79A to assess tumor cell proliferation, stromal cell activation, endothelial cell proliferation, and immune cell viability, respectively (Figure 5E). Histological assessment of TPCS at days 1, 4, 8, and 11 revealed that TME characteristics were maintained throughout the 11-day culture period: dense tumor tissue was highlighted via H&E stain, alongside an αSMA^+^ stroma, and a VCAM-1^+^ endothelium, which developed throughout the culture period. Proliferative Ki-67^+^ cells were still evident in the tissue at day 11, alongside CD3^+^ T cells, F4/80^+^ macrophages, and CD79A^+^ B cells, which were retained in TPCS harvested after 11 days.

To provide utility as a therapeutic screening platform capable of assessing a wide range of compounds, the TPCS system was miniaturized to enable medium-throughput screening of drugs. TPCS 3 mm in diameter were generated as opposed to the 8 mm tissue slices described previously,^14^ and were subsequently cultured in either static 96-well culture plates or a 96-well version of the Bioreactor for a total of 9 days (Figure 5F). The metabolic activity and cytotoxicity of rocked and static 3 mm TPCS were determined via resazurin assay and LDH assay, respectively, revealing that, akin to the 12-well TPCS, 3 mm TPCS do not require the rocked Bioreactor system to maintain tissue viability (Figures 5G, H). This considerably simplifies the platform for adoption by other laboratories that lack access to a bioreactor system.

We next developed a non-destructive method to allow longitudinal monitoring of TPCS viability, with the purpose of enabling dynamic drug screening. Hep-53.4 cells were transfected with a custom vector inducing the expression of secreted luciferase (Supplemental Figure S1A, Supplemental Digital Content 1, http://links.lww.com/HC9/B979), which allows for longitudinal monitoring of tumor cell viability by analyzing media samples that are replaced daily. Assessing media samples collected from 3 mm “SecLuc” TPCS in static culture demonstrated that increased levels of luciferase were secreted from the tissue as the culture period progressed, with an exponential increase in luciferase secretion between days 7 and 11, indicative of TPCS viability and cancer cell proliferation (Figure 5I).

These further innovations to the ex vivo TPCS model therefore provide a simplified and miniaturized TME culture system, whereby a greater number of therapeutic avenues can be explored using the same quantity of tumor tissue, and with a method of assessing the dynamic range of TPCS.

### Developing a medium-throughput TPCS screening platform to identify novel therapies

**FIGURE 6:**
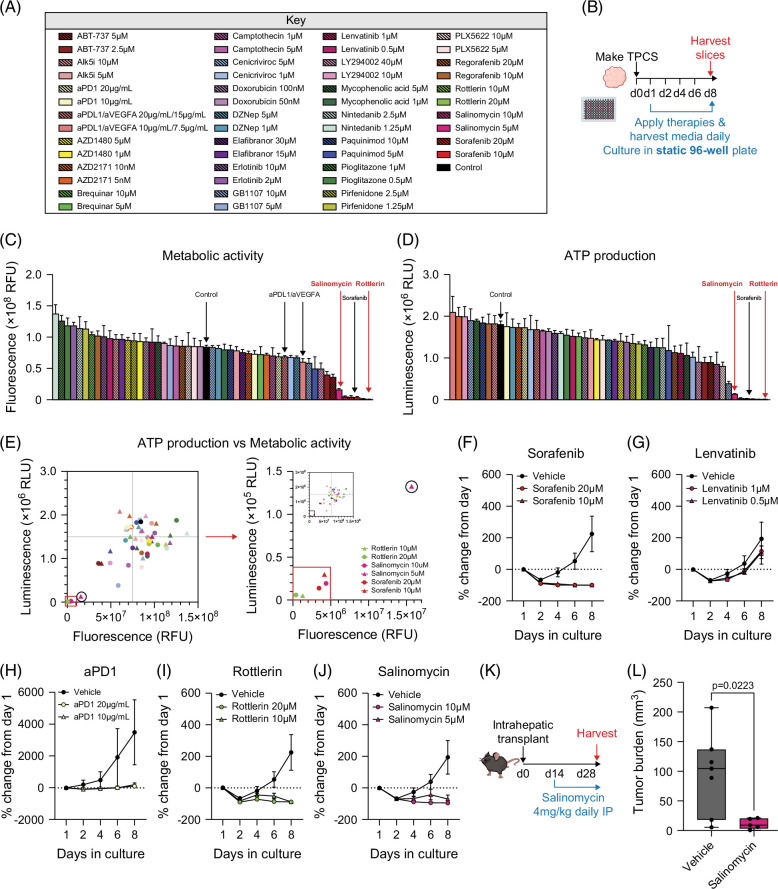
Developing a medium-throughput therapeutic screening platform. (A) The key details of the panel of 26 therapeutic molecules used in the TPCS drug screen, and the concentrations at which they were applied. (B) Schematic shows the timeline of 3 mm TPCS generation and subsequent 8-day culture period in static 96-well culture plates, with 26 drugs from the screening panel applied from day 1 onward. (C, D) Graphs show day 8 (C) metabolic activity and (D) ATP production from TPCS cultured with 26 therapies from the screening panel applied at 2 doses, measured via resazurin assay and CellTiter-Glo assay, respectively. Data are mean ± SEM for N=6 TPCS from N=5 tumors. (E) The graph shows a correlation between ATP production and metabolic activity, measured by CellTiter-Glo assay and resazurin assay, respectively, from TPCS cultured with 26 therapies from the screening panel applied at 2 doses, with emphasis on rottlerin, salinomycin, and sorafenib. (F–J) Graphs show luciferase assay data from TPCS cultured with vehicle control and 2 doses of (F) sorafenib, (G) lenvatinib, (H) anti-PD1, (I) rottlerin, and (J) salinomycin. Data are mean ± SEM from N=6 TPCS from N=5 tumors. (K) The schematic shows the timeline of intrahepatic transplant of Hep-53.4 cells to generate orthotopic tumors, followed by therapeutic intervention at day 14 with salinomycin (4 mg/kg) or vehicle control via daily intraperitoneal injection, before tumors were harvested at day 28. (L) The graph shows tumor burden of Hep-53.4 tumors treated with vehicle control or salinomycin (4 mg/kg). Data are mean ± SEM from up to N=7 mice per treatment group. Abbreviation: TPCS, tumor precision-cut slices.

We next assessed the utility of the miniaturized SecLuc-TPCS model to determine efficacies of potential therapeutic molecules. The panel of compounds selected for the screen included molecules known to target HCC, such as sorafenib, lenvatinib, and anti-PD1, as well as compounds with the potential to modify relevant pathologies such as fibrosis and steatosis in the HCC-TME, and molecules capable of initiating apoptosis in cancer cells (Figure 6A). After initially assessing the efficacy of the compounds against 2D and 3D Hep-53.4 cells (Supplemental Figures S4A–D, Supplemental Digital Content 1, http://links.lww.com/HC9/B979), miniaturized TPCS were generated from Hep-53.4 SecLuc tumors and cultured with 2 doses of each molecule from day 1 to day 8 (Figure 6B). Whilst many of the treated TPCS appeared viable at day 8, the first-line HCC therapy sorafenib unsurprisingly caused a drastic reduction in metabolic activity and ATP production comparable to two additional drugs in the screen, the antibiotic salinomycin and the PKC inhibitor rottlerin (Figures 6C, D). Rottlerin has previously exhibited anticancer activity by inducing apoptosis and inhibiting cell growth, migration and invasion, and has been observed to upregulate the tumor suppressor DDX3 in HCC cells.^21^^,^^22^ Salinomycin selectively targets cancer stem cells and exerts anticancer effects by initiating ferroptosis via the accumulation of iron in lysosomes.^23^ Comparing the ATP production and metabolic activity of TPCS in the drug screen identified a positive correlation between the 2 readouts, with sorafenib, rottlerin and salinomycin substantially attenuating each measure of tissue viability compared to the remaining compounds (Figure 6E).

Implementing the secreted luciferase “SecLuc” system enabled TPCS viability to be dynamically examined throughout the time course of the therapeutic screen and avoided the use of unpaired media samples. In addition to highlighting that most therapies resulted in lower secreted luciferase levels compared to the controls at end-point (day 8) (Supplemental Figure S4E, Supplemental Digital Content 1, http://links.lww.com/HC9/B979), the luciferase read-out provided a dynamic range illustrating that vehicle-treated TPCS secreted more luciferase as the culture period progressed from day 2 to day 8 (Figures 6F–J). Although the first-line TKI sorafenib completely suppressed luciferase secretion from TPCS, the first-line TKI lenvatinib failed to reduce levels of secreted luciferase compared to the vehicle, potentially due to lenvatinib possessing greater potency against the EGF receptor and VEGF receptors alongside the lack of a mature vasculature in TPCS (Figures 6F, G).^20^^,^^24^ Investigating TPCS response to immunotherapy demonstrated that anti-PD1 had a cytostatic effect, such that secreted luciferase levels were unchanged throughout the culture period (Figure 6H). Comparable to metabolic activity and ATP production, both salinomycin and rottlerin successfully reduced the levels of secreted luciferase compared to the vehicle controls, as well as the baseline day 1 luciferase levels indicative of tumor cell killing (Figures 6I, J). An additional 8 therapeutic molecules were also screened in the miniaturized TPCS system in a second drug screen (Supplemental Figures S4F–H, Supplemental Digital Content 1, http://links.lww.com/HC9/B979), identifying the multi-kinase inhibitor Rigosertib and a PFKFB3 inhibitor as promising anti-HCC candidate drugs.

The antibiotic salinomycin has previously been reported to possess anticancer properties, and has demonstrated efficacy in targeting murine orthotopic HepG2 tumors without reported adverse effects.^25^ Salinomycin was therefore selected to target Hep-53.4 tumors in vivo, so as to validate the capability of the TPCS model for identification of efficacious anticancer compounds prior to conducting costly in vivo drug testing experiments. To determine if the cytotoxic effect observed in TPCS is specific to the tumor and does not affect the surrounding non-tumor liver tissue, static TPCS and PCLS cultured in the rocked Bioreactor system were treated with salinomycin to assess impacts on metabolic activity (Supplemental Figure S5A, Supplemental Digital Content 1, http://links.lww.com/HC9/B979). Resazurin assays performed on TPCS across an 8-day period illustrated that 10 μM and 5 μM salinomycin severely impacted viability by day 5 and day 7, respectively in a similar manner to 20 μM sorafenib which was used as a positive control, while the vehicle treated TPCS remained viable until day 8 (Supplemental Figure S5B, Supplemental Digital Content 1, http://links.lww.com/HC9/B979). In contrast, PCLS treated with salinomycin remained viable until day 3, comparable to vehicle treated PCLS, before all PCLS became unviable in culture at day 4 and independently of salinomycin treatment (Supplemental Figure S5C, Supplemental Digital Content 1, http://links.lww.com/HC9/B979). After determining that salinomycin did not exert obvious cytotoxic effects on non-tumor liver tissue, mice with implanted Hep-53.4 tumors were treated with 4 mg/kg salinomycin (Figure 6K). This resulted in a significant reduction in tumor burden compared to vehicle-control treated tumors, with macroscopic tumors just about visible following salinomycin treatment (Figure 6L), while there was a negligible impact on liver weight, no significant difference in serum aspartate aminotransferase levels and no obvious histological evidence of hepatotoxicity (Supplemental Figures 5D–F, Supplemental Digital Content 1, http://links.lww.com/HC9/B979).

Establishing salinomycin as a drug capable of targeting in vivo Hep-53.4 tumors following its identification in the therapeutic screen highlights the potential of the TPCS platform to identify novel HCC therapies. This could avoid high costs, animal suffering, and unnecessary use of large numbers of animals to assess compounds that do not possess potent anticancer activity.

## DISCUSSION

The treatment landscape for HCC is advancing at a considerable pace, evolving over the past 2 decades from single-agent TKIs to tailored combination therapies and the integration of immunotherapy into HCC clinics as front-line systemic therapies. Resultant of new insights into the biology and immunology of HCC, we can expect new therapeutic targets to continue to emerge and inform the design of novel drugs and combination strategies. Experimental mouse models of HCC are currently the mainstay preclinical platform for determining the likely efficacy of novel drugs and combination therapies. While such models can be powerful translational tools, they have inherent limitations, not least high costs and incurred suffering of high numbers of animals. In addition, in vivo mouse models of most cancers do not readily lend themselves to “at-scale” drug screens or for the robust testing of experimental therapies, where determining the effective dosage and timing of drug administration can be critical in achieving an optimal therapeutic outcome.^26^ With HCC as our paradigm disease, we have produced a viable and accessible solution to the limitations of in vivo oncology studies in mice by optimizing the ex vivo culture of live TPCS, including their miniaturization to 96-well culture scale and dynamic quantification of tumor cell viability.

Tumor slice cultures have previously been reported to maintain the architecture and diverse cellular constituents of the TME.^27^ Tumor slice cultures are reported to maintain T cell populations of the original tumor with potential for immunotherapy investigations.^27^ Both human and mouse tumor slices have been established for a variety of tumors; however, their application for drug screening is yet to be fully realized. Concerning HCC, Jagatia et al^15^ recently described the use of human liver tumor slices as a preclinical platform for primary liver cancer samples. This latter study described maintenance of HCC-derived and cholangiocarcinoma-derived slice cultures for up to 8 days with retention of proliferating tumor cells, stromal cells, and continued presence of intratumoral CD3^+^ and CD45^+^ leukocytes. The authors also validated HCC slices as a model for preclinical testing of TKIs and anti-PD1. However, despite this advance, there are considerable limitations on the wider utility of human tumor slices, including the availability of fresh human tumor tissue, which requires a local surgical oncology unit, timely acquisition of the tissues from operating theaters, and clinical pathology assessment of tissues prior to their transfer to the laboratory. Furthermore, preclinical oncology models that are to be utilized for drug development require standardization to overcome inherent variables that influence the biology of the tumor slices and therapeutic response, such as intratumor and intertumor heterogeneity. These variables are extremely challenging to overcome for many human solid tumors, including HCC. Our rationale was therefore to combine the standardization and ease of accessibility of mouse modeling with optimization and scaling of ex vivo mouse HCC tumor slices (HCC-TPCS) to produce a 3-dimensional TME platform that can be used in most laboratories, including in an industry setting where access to human tumor tissue can be rare and unpredictable.

As with human tumor slices, TPCS from the orthotopic HCCs retained proliferative tumor cells, stromal cells, plus a variety of immune cells throughout an extended culture period of 11 days, although neutrophils were lost from the tumor ecosystem due to their short lifespan. Whilst an isolated TPCS cannot receive a continual supply of recruited immune cells from the circulation, supplementing the tissue culture with immune cells isolated from blood offers a compromise and a method of modeling immune cell recruitment. Given the viability of TPCS was maintained for up to 11 days, further extending the culture period beyond this time may be possible if specific investigations require it. Regarding immune checkpoint blockade, we were able to demonstrate that resident CD8^+^ T cells expanded and expressed Ki-67^+^, indicative of induced proliferation within the cultured TME. As we have previously described the requirement of a rocked bioreactor system for optimal maintenance of rat and human liver slices^14^ we initially cultured HCC-TPCS under these conditions, however subsequent experiments indicated no advantage of the bioreactor for tumor slices. To allow for dynamic monitoring of tumor cell numbers, we generated Hep-53.4 tumor cells expressing a secreted luciferase and employed these cells for orthotopic tumor and TPCS generation, enabling the kinetics of drug cytotoxicity to be monitored by measurement of luciferase activity in the culture media. The second modification was to miniaturize the platform to 96-well scale, enabling us to screen 26 therapeutic agents at 2 doses with the inclusion of appropriate controls using a total of 5 ex vivo tumors. A similar screen carried out in vivo would require 520 test mice in addition to vehicle and antibody controls when using 10 mice per group, this highlighting the potential for ex vivo TPCS to dramatically reduce animal usage for oncology as well as enabling at-scale drug screening in an intact murine TME. The screen successfully identified 2 drugs (rottlerin and salinomycin) not currently used in the HCC clinic, of which salinomycin was subsequently validated as effective for reducing tumor burden in vivo. These TPCS adaptations therefore provide the opportunity for real-time monitoring of drug screens at medium-throughput scale and, most importantly, in the context of a live and anatomically faithful TME that maintains key cell types. Of note, TPCS can be processed downstream of a drug study for flow cytometry, live cell imaging, generating single cell transcriptomics data, and producing spatial omics data which if combined will exploit the TME architecture of tumor slices to report effects of therapeutic molecules with a more holistic approach than can possibly be achieved with simpler 2D or 3D cell culture systems, including organoids. It will also be possible to genetically modify the Hep-53.4 cell line, for example, by CRISPR/Cas9 technology, to allow drugs to be screened in the context of mutations commonly associated with human HCC. This may improve our understanding of the mechanisms by which oncology agents exert beneficial effects, and also predict toxicities and unwanted influences on the viability and phenotypes of tumor-associated immune cells. Of the 3 cell lines employed to initially develop orthotopic tumors, Hep-53.4 was the only cell line to reliably exhibit a high engraftment rate; a partial hepatectomy has previously been shown to promote the growth of orthotopically transplanted H22 cells, and while more invasive may therefore provide a method of improving HCC engraftment with a wider range of cell lines.^28^ These insights can then be employed to improve the design of future cancer therapeutics.

In summary, we report a dynamic 96-well scale murine HCC-TME platform for oncology biology and drug development purposes that can be employed by academic and industry laboratories that have access to an animal unit, appropriate ethical and regulatory approvals, and standard cell culture facilities. As we were able to routinely generate a full 96-well plate of TPCS from a single tumor, the platform delivers an estimated 98.7-fold reduction (assuming n=4 TPCS per group for 11 compounds and 1 vehicle control) in use of animals and avoids exposing large numbers of mice to experimental drugs which may exert unpleasant adverse effects. We envisage the platform integrating downstream of higher-throughput human cell culture systems, such as patient-derived organoids, to validate drug efficacies and explore therapeutic mechanisms in the intact TME, replacing the need for in vivo preclinical studies as part of the drug development process. The adaptations we report could also be applied to other primary and secondary tumors of the liver. Moreover, with advancing improvements in the engineering of humanized mice and their combination with implanted patient tumor tissues, the TPCS platform can be further developed to deliver personalized oncology at a scale that allows for examination of complex combinatorial drug dosing and sequencing.

## Supplementary Material
