# Supplementary material for: Precision-cut tumor slices for modeling hepatocellular carcinoma enable at-scale drug screening
Source: Hepatol Commun. 2025 May 16;9(6):e0706. doi: 10.1097/HC9.0000000000000706 (PMC12088631; doi:10.1097/HC9.0000000000000706)
Supplement: Supplementary file 1 [file hc9-9-e0706-s001.pdf]

Supplemental Digital Content (SDC)

Methods

Peripheral blood mononuclear cell (PBMC) isolation

300 µl mouse blood was collected in a EDTA Vacutainer tube following exsanguination under terminal anaesthesia by severance of the inferior vena cava. 2.5 ml Histopaque-1077 (Sigma-Aldrich) was layered on top of 2.5 ml Histopaque-1119 (Sigma-Aldrich). The blood was diluted to a total of 6 ml in PBS and carefully layered on top of the Histopaque-1077 prior to centrifugation at 700g for 30 minutes with no brake. The top band containing mononuclear cells was isolated from the separation and stained with CellTracker™ Green CMFDA (Thermo Fisher) as per the manufacturers' instructions.

Transfection

Stable transfections were performed on Hep-53.4 cells using a custom pNL(sNLuc/CMV/NeoR) (**Supplemental Figure 1**) expression vector using the Lipofectamine 3000 Transfection Reagent kit (Thermo Fisher). 100,000 cells were seeded in each well of a 6-well plate. The following day, a transfection complex comprised of 250 µl Opti-MEM™ (Thermo Fisher), 5 µl P3000 Reagent, 7.5 µl Lipofectamine 3000 Reagent and 2.5 µg plasmid DNA was mixed and left for 10 minutes to enable DNA-lipid formation. The cell culture media was refreshed, and the transfection complex was added to the cells in a drop-wise manner. The following day, the media was removed from all cells and replaced with selection media containing 1 mg/ml G418 disulfate salt (Sigma). The cells remained in selection media until all cells in a control well had visibly died. Transfected cell lines were cultured in selection media approximately once every two months to prevent an expansion of WT cells.

Tissue culture treatments

All TPCS and PCLS were cultured in supplemented Williams' Medium E alone for 1 day to allow the tissue to stabilise. To investigate TKI response, TPCS and PCLS 8 mm in diameter were cultured in BioR plates in the Bioreactor and treated with sorafenib (10 µM – 20 µM) (Tocris), lenvatinib (0.25 µM – 1.0 µM) (Selleckchem) or DMSO control media for a further 3 days after the rest period.

To model immunotherapy responses, TPCS 8 mm in diameter were cultured in BioR plates in the Bioreactor, and treated with 20 µg/ml Ultra-LEAF™ Purified anti-mouse CD279 (PD-1) (Biolegend) or rat IgG2a, κ isotype control (Biolegend) from day 2 until the TPCS were harvested at day 4.

To validate the static culture and scaling of TPCS, TPCS 8 mm and 3 mm in diameter were cultured in standard 12-well (8 mm) and 96-well (3 mm) culture plates with Williams' Medium E for up to 11 days.

To screen a panel of 26 compounds, TPCS 3 mm in diameter were cultured in static 96-well culture plates and treated with two doses of each compound or controls for a further 7 days after the rest period.

To investigate salinomycin toxicity PCLS and TPCS 3 mm in diameter were cultured in BioR plates in the Bioreactor with DMSO control, salinomycin (5 µM – 10 µM) or sorafenib (20 µM) for a further 3 days (PCLS) or 7 days (TPCS) after the rest period.

To supplement TPCS with additional PBMCs, approximately 300,000 PBMCs labelled with CellTracker™ Green CMFDA (Thermo Fisher) were added to the surface of a TPCS and cultured for a further 24 hours. The TPCS was fixed in 4% paraformaldehyde (PFA) solution for 30 minutes, then stained with 10 µg/ml Hoechst 33342 for a further 30 minutes. The tissue was then washed in PBS and transferred onto a microscope slide inside a gene frame and mounted with VECTASHIELD© Antifade Mounting Medium (Vector Laboratories).

Multiphoton imaging

Multiphoton imaging was performed using a Zeiss LSM880 NLO Multiphoton microscope coupled to a Coherent Chameleon Discovery pulsed laser. z-stack images were captured using a 25x / 0.8 NA LD-LCI-Plan Apochromat multi immersion lens (configured for glycerol immersion), exciting the sample with 760 and 920 nm for Hoechst and CellTracker Green, capturing fluorescence emission with Shortpass (485 nm) and Bandpass (500-550 nm) filters in front of NDD PMT and GaAsP PMT detectors respectively. To assess larger areas, each acquisition was performed as a tilescan and merged in the acquisition software (Zen Black v2.3). Labelled PBMC migration into the TPCS were ascertained from measuring the depth of green fluorescence on MIP projected orthogonal views of the z stacks.

Colorimetric assays

CyQUANT™ LDH Cytotoxicity Assay (Thermo Fisher) and AST Activity Assay (Sigma-Aldrich) were performed as per the manufacturers' instructions.

Enzyme linked immunosorbent assay (ELISA)

Sandwich ELISA quantification for mouse albumin (ab210890, Abcam) was performed according to the manufacturers' instructions on culture media samples harvested daily from PCLS.

Resazurin assay

4.5 mM resazurin stock solution was diluted in Williams' Medium E to a working concentration of 450 µM. Each tissue slice was incubated in a 96-well plate with 100 µl of the working resazurin solution for 1 hour at 37°C with 5% CO_2. 100 µl of the working resazurin solution was used as a negative control. After 1 hour,

the solution was transferred to an opaque 96-well plate (Greiner) and the fluorescence was measured on a Filtermax F5 multi-mode plate reader, ex 535 nm and em 595 nm.

Luminescent assays

Levels of secreted luciferase in the TPCS culture media from SecLuc transfected HCC cells was determined using the Nano-Glo® Luciferase Assay System (Promega). Nano-Glo® Luciferase Assay Reagent was prepared as per the manufacturers' instructions. The media samples were combined in a 1:1 ratio with the Nano-Glo® Luciferase Assay Reagent in an opaque 96-well plate (Greiner) and left for at least 3 minutes for the reaction to take place. The luminescence values were then measured using Filtermax F5 multi-mode plate reader. Cell Titer-Glo® 3D Cell Viability Assay (Promega) was performed as per the manufacturers' instructions.

RNA isolation, cDNA synthesis and PCR

The QIAGEN RNeasy Mini Kit (QIAGEN) was used to extract RNA from tissue as per the manufacturers' instructions. 1 µg RNA was treated with DNase (Promega) and cDNA was synthesised via incubation with random hexamer primer (Promega) and M-MLV reverse transcriptase (Promega). Real-time PCR was carried out using SYBR Green JumpStart Taq ReadyMix (Sigma) as per the manufacturers' instructions with primers previously described (1).

Histology and immunohistochemistry

Frozen 10 µm thick tissue sections were stained with Oil Red O, and formalin-fixed, paraffin-embedded 5 µm thick tissue sections were stained with haematoxylin and eosin using established protocols. To perform immunohistochemistry, sections were first deparaffinised and endogenous peroxidase activity was blocked with 0.6% hydrogen peroxide/methanol solution. Antigen retrieval was performed using either antigen unmasking solution (Vector Laboratories) for αSMA (1:1000; F3777; Sigma-Aldrich), Active Caspase-3 (1:400; 9661; Cell Signaling), Cytokeratin 19 (1:250; ab85632; Abcam), Ly6G (1:200, ab210204; Abcam) and PCNA (1:3000; ab18197; Abcam), or 1 mM Tris-EDTA (pH 9.0) for CD3 (1:200; MCA1477; Bio-Rad), CD79A (1:5000; ab300150; Abcam), Cytokeratin 18 (1:800; ab181597; Abcam), F4/80 (1:800; 70076; Cell Signaling), HIF-1α (1:200; 48085S; Cell Signaling), Ki-67 (1:10,000; 14-5698-82; Thermo Fisher) or VCAM-1 (1:200; 32653S; Cell Signaling). The Avidin/Biotin Blocking Kit (Vector Laboratories) was used to block endogenous avidin and biotin, followed by blocking of non-specific binding with Normal Goat Serum (Vector Laboratories) for 45 minutes. Tissue sections were then incubated with primary antibodies at the relevant dilutions overnight at 4°C. The slides were washed, and sections were incubated with biotinylated goat anti-rabbit (1:600; Vector Laboratories), biotinylated goat anti-rat (1:200; Serotec) or biotinylated goat anti-fluorescein (1:300; Vector Laboratories) secondary antibodies for 45 minutes. Slides were washed and incubated with Vectastain Elite ABC HRP Reagent (Vector Laboratories) for 30 minutes. Staining was

developed using DAB peroxidase substrate kit (Vector Laboratories), followed by counterstaining with Mayer's haematoxylin. Sections were then dehydrated and mounted in Pertex Mounting Medium (Cell Path). Terminal deoxynucleotidyl transferase-mediated dUTP nick end (TUNEL) labelling was performed using the TUNEL Assay Kit – HRP-DAB (Abcam) as per the manufacturer's instructions. Tissue sections were analysed at 20x magnification using a Nikon Eclipse Ni-U microscope and NIS-Elements BR analysis software. A minimum of 12 non-overlapping fields were analysed from *in vivo* tissue sections, and a minimum of 6 non-overlapping fields were analysed from PCLS and TPCS due to the smaller available area of tissue.

Hyperion imaging mass cytometry

Hyperion Imaging Mass Cytometry (IMC) was performed on formalin-fixed paraffin-embedded (FFPE) 5 µm thick tissue sections. Slides were first deparaffinised and rehydrated by passing the sections through clearene, followed by 100%, 90%, 70% and 50% ethanol solution for 5 minutes each. Sections were then washed in deionised water for 5 minutes, before heat-mediated antigen retrieval was performed using Tris-EDTA solution at pH 9.0. The sections were allowed to cool and then washed in PBS for 5 minutes. A ring was drawn around each tissue section with a hydrophobic pen and non-specific binding was blocked with 3% BSA/PBS for 45 minutes. 200 µl of metal-conjugated CD8 (rabbit monoclonal, clone EPR21769, Abcam) and Ki-67 (Rat monoclonal, clone SolA15, Thermo Fisher) primary antibodies was then added to each section in 0.5% BSA/PBS and incubated overnight at 4°C. CD8 and Ki-67 primary antibodies were labelled with 164 Dy and 163 Dy metal isotopes respectively using the Maxpar® X8 Multimetal Labeling Kit—40 Rxn (201300, Standard Biotools). The sections were washed in Tris-Buffered Saline + 0.1% Tween (TBS-T) followed by two consecutive washes in PBS. The sections were then incubated for 30 minutes with 125 µM (193lr) Intercalator at a dilution of 1:400. The sections were washed in ultra-pure water for 5 minutes and subsequently air-dried at room temperature. A region of interest (ROI) was selected around the HCC spheroid and the tissue was ablated by the Hyperion Fluidigm Imaging System.

Supplemental Figures and Figure Legends

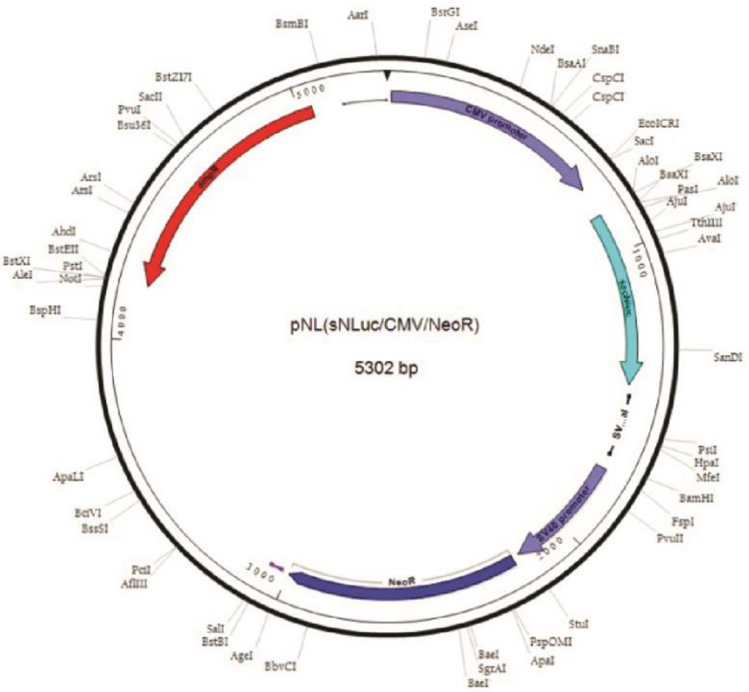

Supplemental Figure 1

Vector map of custom plasmid pNL(sNLuc/CMV/NeoR) (Promega) inducing the expression of secreted NanoLuc luciferase.

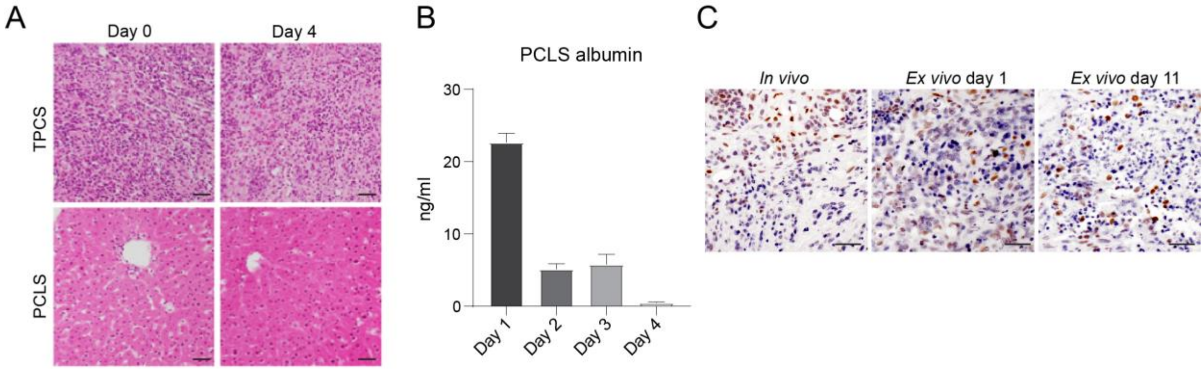

Supplemental Figure 2

(A) Representative images of H&E-stained TPCS and PCLS at day 0 and day 4 following culture in a rocked Bioreactor platform. (B) Graph shows concentration of soluble albumin secreted from PCLS cultured for 4 days in the rocked Bioreactor platform. Data are mean ± s.e.m. from up to N=20 paired PCLS. (C) Representative images of HIF-1α-stained tumour and TPCS at day 1 and day 11. Scale bars: 50 µm.

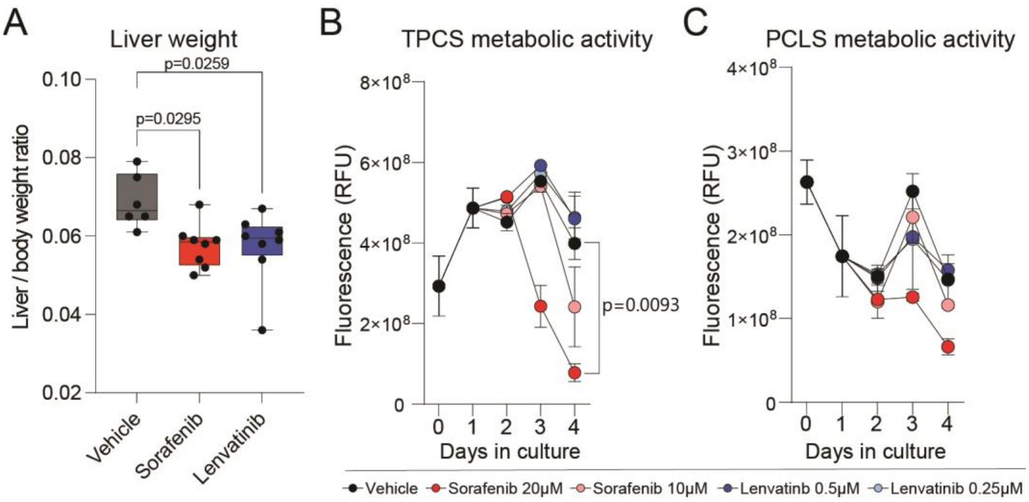

Supplemental Figure 3

(A) Graph shows liver to body weight ratio of mice with Hep-53.4 orthotopic tumours following treatment with vehicle control, sorafenib (45 mg/kg) or lenvatinib (10 mg/kg). Data are mean ± s.e.m. from up to N=8 mice per treatment group. (B-C) Graphs show metabolic activity of (B) TPCS and (C) PCLS cultured for 4 days with vehicle control, sorafenib (10 µM – 20 µM) or lenvatinib (0.25 µM - 0.5 µM), measured via resazurin assay. Data are mean ± s.e.m. from up to N=3 TPCS/PCLS per treatment group and time point.

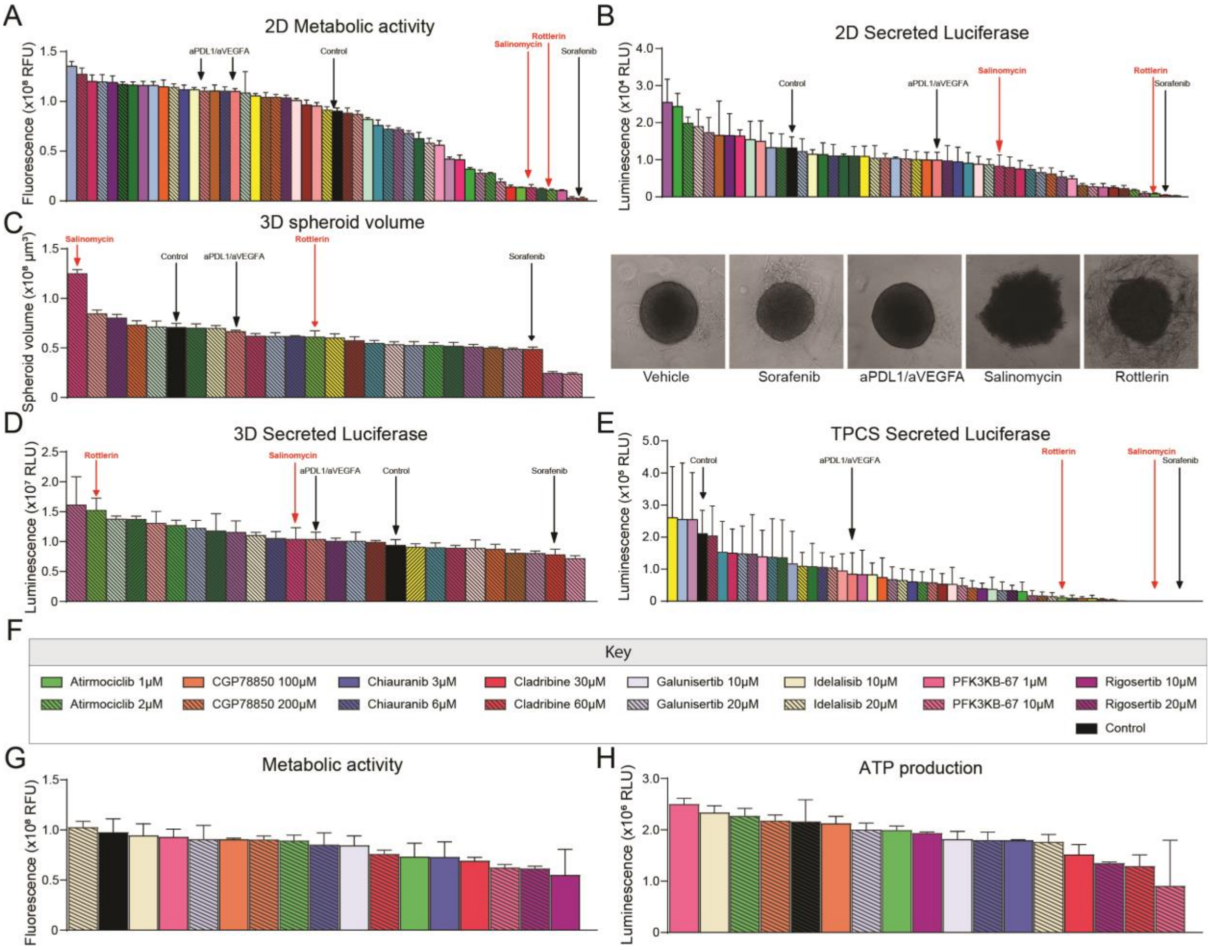

Supplemental Figure 4

(A-B) Graphs show day 4 (A) metabolic activity measured via resazurin assay and (B) secreted luciferase levels from 2D Hep-53.4 cells cultured with therapies from the screening panel applied at two doses. Data are mean ± s.e.m. for N=3 wells per treatment. (C-D) Graph and representative images show day 4 (C) volume and (D) secreted luciferase levels of 3D Hep-53.4 spheroids cultured with therapies from the screening panel applied at one dose. Data are mean ± s.e.m. for N=4 spheroids per treatment. (E) Graph shows day 8 secreted luciferase levels from TPCS cultured with 26 therapies from the screening panel applied at two doses. Data are mean ± s.e.m. for N=6 TPCS from N=5 tumours. (F) The key details the panel of 8 therapeutic molecules used in a second TPCS drug screen and the concentrations that they were applied at. (G-H) Graphs show day 8 (G) metabolic activity and (H) ATP production of TPCS in second therapeutic screen performed with 8 additional compounds applied at two doses. Data are mean ± s.e.m. for N=3 TPCS from N=2 tumours.

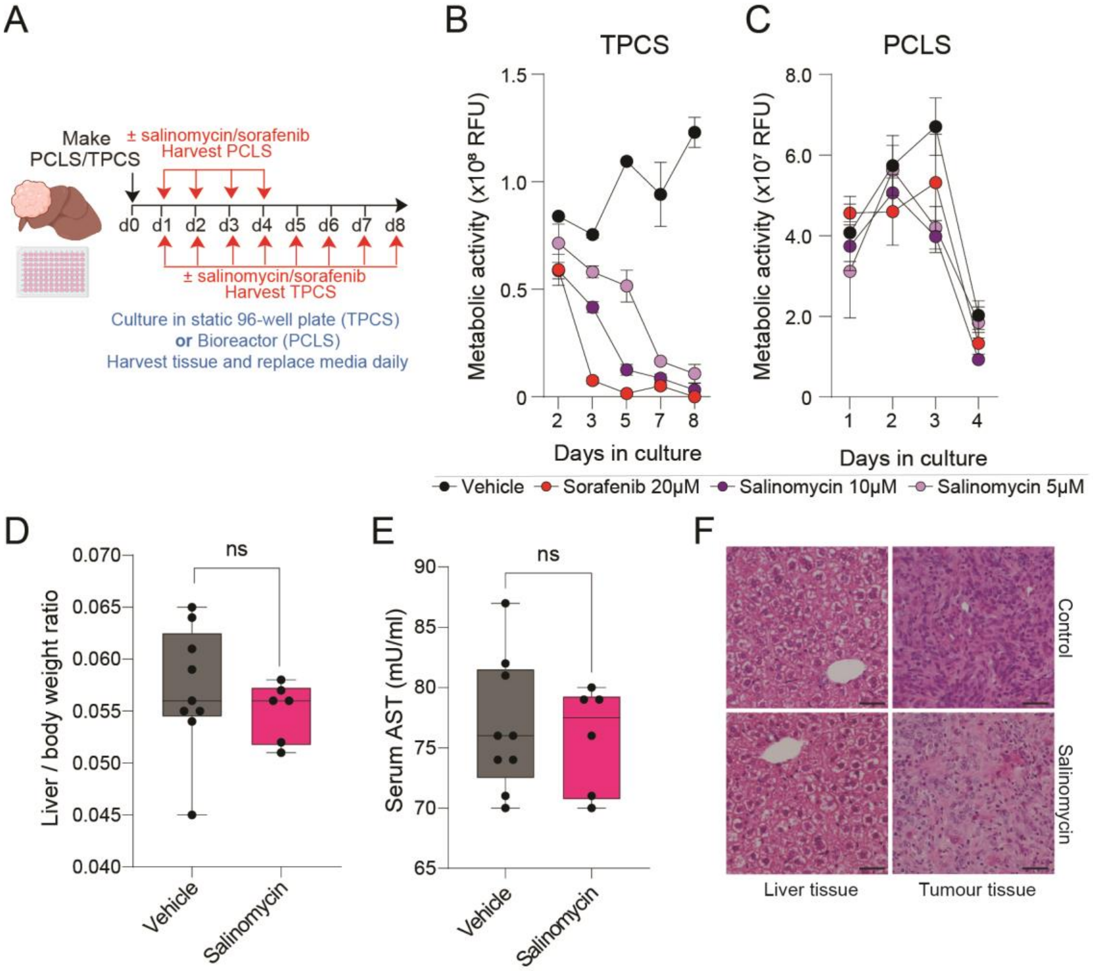

Supplemental Figure 5

(A) Schematic shows the timeline of PCLS and TPCS generation and subsequent culture period of 4 and 8 days respectively, cultured in the presence or absence of sorafenib (5 µM) or salinomycin (5 µM – 10 µM). (B-C) Graphs show metabolic activity of (B) TPCS and (C) PCLS cultured for 8 and 4 days respectively with vehicle control, sorafenib (20 µM) or salinomycin (5 µM – 10 µM), measured via resazurin assay. Data are mean ± s.e.m. from up to N=4 TPCS/PCLS per treatment group and time point. (D-E) Graphs show (D) liver

to body weight ratio and (E) serum AST levels of mice with Hep-53.4 orthotopic tumours following treatment with vehicle control or salinomycin (4 mg/kg). Data are mean ± s.e.m. from up to N=9 mice per treatment group. (F) Representative images of H&E-stained livers and tumours of mice treated with vehicle control or salinomycin (4 mg/kg). Scale bars: 50 µm.

References

1. Leslie J, Macia MG, Luli S, Worrell JC, Reilly WJ, Paish HL, et al. c-Rel orchestrates energy-dependent epithelial and macrophage reprogramming in fibrosis. Nat Metab. 2020;2(11):1350-67.
